# Machine Translation for Multilingual Cancer Patient Education: Bridging Languages, Navigating Challenges

**DOI:** 10.1007/s13187-024-02438-5

**Published:** 2024-04-23

**Authors:** Aaron Lawson McLean, Tui Lin Yen

**Affiliations:** 1 Department of Neurosurgery, Jena University Hospital – Friedrich Schiller University Jena, Jena, Germany; 2 Comprehensive Cancer Center Central Germany, Jena, Germany; 3 Department of Education, China Medical University Hospital, Taichung, Taiwan; 4 School of Medicine, College of Medicine, China Medical University, Taichung, Taiwan

**Keywords:** Machine translation, Healthcare equity, Multilingual patient education, Communication barriers, Digital healthcare divide, AI ethics

## Abstract

This commentary evaluates the use of machine translation for multilingual patienteducation in oncology. It critically examines the balance between technologicalbenefits in language accessibility and the potential for increasing healthcare disparities.The analysis emphasizes the need for a multidisciplinary approach to translation thatincorporates linguistic accuracy, medical clarity, and cultural relevance. Additionally, ithighlights the ethical considerations of digital literacy and access, underscoring theimportance of equitable patient education. This contribution seeks to advance thediscussion on the thoughtful integration of technology in healthcare communication,focusing on maintaining high standards of equity, quality, and patient care.

The recent publication in the Journal of Cancer Education by Ugas, Giuliani, and Papadakos titled “When is good, good enough? On considerations of machine translation in patient education” provides a critical examination of the burgeoning role of machine translation in healthcare, specifically in the context of patient education materials for non-English-speaking cancer patients [1]. The authors adeptly highlight the substantial barriers faced by individuals with limited English proficiency (LEP) in accessing healthcare information, the prohibitive costs of human translation for healthcare institutions, and the potential for machine translation to serve as a pragmatic solution within these constraints. However, this discourse also raises several complex issues that merit further exploration, especially concerning the potential for machine translation to perpetuate existing inequities and the importance of nuanced understanding in healthcare communication.

Firstly, while machine translation technologies have indeed made significant strides in accuracy and reliability, their application in healthcare settings warrants a cautious approach. The essence of patient education is not merely to inform but to empower patients towards greater self-efficacy in managing their health. The nuanced complexities of medical terminology, the cultural context of health beliefs, and the individual patient’s cognitive and emotional state are all critical components of effective patient education. Machine translation, despite its advancements, lacks the ability to fully grasp these subtleties [2]. The risk of misinterpretation is not merely a technical issue but a matter of patient safety and quality of care. Miscommunications in healthcare settings can lead to non-adherence to treatment plans, increased anxiety, and ultimately, worse health outcomes.

Moreover, the reliance on machine translation without adequate oversight and quality control mechanisms can inadvertently perpetuate systemic biases. Language, as a social construct, carries with it the nuances of cultural, socioeconomic, and historical contexts. Automated translation systems, trained on existing datasets, may replicate and amplify biases present in these data [3]. For instance, translations could inadvertently reinforce stereotypes or omit culturally specific health beliefs and practices, thus alienating the very populations they aim to serve. The move towards machine translation raises ethical questions about whose voices are prioritized and whose are marginalized in the quest for linguistic accessibility.

The proposition of a hybrid model, combining machine translation with human post-editing, is a step towards mitigating these risks [4]. However, this approach also underscores the indispensable value of human expertise in translation, particularly for materials with significant clinical ramifications. It is crucial that these human translators are not only linguistically skilled but also culturally competent, with a deep understanding of the health beliefs, practices, and nuances of the target population. This emphasizes the need for a multidisciplinary approach to translation that integrates linguistic, medical, and cultural expertise.

The implementation of machine translation in healthcare also raises significant questions about digital literacy and access. The shift towards web-based patient education materials assumes a level of digital access and literacy that may not be universal. Populations with limited digital literacy or access, who are often overlapping with those requiring translation services, may find themselves further marginalized [5]. Thus, while addressing language barriers, we must also be mindful not to exacerbate the digital divide.

In conclusion, while the pragmatism of leveraging machine translation in the face of fiscal and logistical constraints cannot be understated, it is imperative that we approach this solution with a critical eye towards the potential for unintended consequences. The goal should not merely be to translate words but to ensure that the essence of patient education—empowerment, understanding, and engagement—is not lost in translation. As we navigate the complexities of language, technology, and healthcare, a collaborative, multidisciplinary approach that centers the needs and perspectives of the diverse patient population is essential. This endeavor is not only a technical challenge but a profound responsibility to uphold the principles of equity, quality, and compassion in healthcare.

## Data Availability

All data generated or analyzed during this study are included in this published article. No additional data are available.
